# Improving follow-up of abnormal cancer screens using electronic health records: trust but verify test result communication

**DOI:** 10.1186/1472-6947-9-49

**Published:** 2009-12-09

**Authors:** Hardeep Singh, Lindsey Wilson, Laura A Petersen, Mona K Sawhney, Brian Reis, Donna Espadas, Dean F Sittig

**Affiliations:** 1 The Center of Inquiry to Improve Outpatient Safety Through Effective Electronic Communication and the Houston VA HSR&D Center of Excellence at the Michael E DeBakey Veterans Affairs Medical Center, VA Medical Center (152) 2002 Holcombe Blvd, Houston, TX 77030, USA; 2 Section of Health Services Research, Department of Medicine, Baylor College of Medicine, Michael E DeBakey Veterans Affairs Medical Center (MEDVAMC), HSR&D Center of Excellence (152) 2002 Holcombe Boulevard, Houston, TX 77030 USA; 3 The Center of Inquiry to Improve Outpatient Safety Through Effective Electronic Communication, University of Texas School of Health Information Sciences and the UT-Memorial Hermann Center for Healthcare Quality & Safety, 6410 Fannin, UPB 1100 Houston, Texas 77030-3006, USA

## Abstract

**Background:**

Early detection of colorectal cancer through timely follow-up of positive Fecal Occult Blood Tests (FOBTs) remains a challenge. In our previous work, we found 40% of positive FOBT results eligible for colonoscopy had no documented response by a treating clinician at two weeks despite procedures for electronic result notification. We determined if technical and/or workflow-related aspects of automated communication in the electronic health record could lead to the lack of response.

**Methods:**

Using both qualitative and quantitative methods, we evaluated positive FOBT communication in the electronic health record of a large, urban facility between May 2008 and March 2009. We identified the source of test result communication breakdown, and developed an intervention to fix the problem. Explicit medical record reviews measured timely follow-up (defined as response within 30 days of positive FOBT) pre- and post-intervention.

**Results:**

Data from 11 interviews and tracking information from 490 FOBT alerts revealed that the software intended to alert primary care practitioners (PCPs) of positive FOBT results was not configured correctly and over a third of positive FOBTs were not transmitted to PCPs. Upon correction of the technical problem, lack of timely follow-up decreased immediately from 29.9% to 5.4% (p < 0.01) and was sustained at month 4 following the intervention.

**Conclusion:**

Electronic communication of positive FOBT results should be monitored to avoid limiting colorectal cancer screening benefits. Robust quality assurance and oversight systems are needed to achieve this. Our methods may be useful for others seeking to improve follow-up of FOBTs in their systems.

## Background

Fewer than 75% of patients with abnormal cancer screening examinations receive follow-up diagnostic care subsequent to the initial screening [1-5]. This inadequate follow-up of abnormal cancer screens compromises the benefits of population-based screening programs [6-9]. For instance, the rate of follow-up for positive fecal occult blood test (FOBT) results in the Veterans Affairs health care system is low; more than 40% of veterans with positive FOBTs may not be receiving timely diagnostic colonoscopies [10,11]. Lack of timely follow-up has also been documented outside the VA system [12,13].

An important, largely preventable but relatively unexplored reason for lack of follow-up is a problem in communication of the positive test result from the laboratory to the clinician who ordered it [14,15]. The use of electronic health records, especially those that utilize such features as automated communication of abnormal results from laboratories to clinicians, can potentially improve follow-up of abnormal cancer screens [16-19]. Electronically "alerting" the ordering provider about an abnormal test result such as positive FOBT can improve the availability of vital information at the point of care [18]. As one of several multifaceted interventions to improve follow-up of positive FOBTs, our institution previously implemented standard operating procedures for the electronic health record's test result communication system [19], including the transmission of a mandatory alert to the patient's clinician for every positive FOBT result. This procedure was expected to reduce breakdowns in communication between the laboratory and clinicians.

A significant increase in timely responses to positive FOBT notifications (defined as a documented response within two weeks of the test) followed implementation of this and several related interventions. However, we found that 40% of automated notifications of FOBT results had no documented response by a treating clinician at two weeks even though all of the patients with these positive FOBT tests were eligible to receive a diagnostic colonoscopy. Our research question was to determine why a large number of FOBT alerts were not followed by clinician response at 2-weeks and to investigate if technical and workflow-related aspects of automated communication in the electronic health record were responsible. We also sought to implement and evaluate a potential solution to the issue(s) we identified.

## Methods

The study was conducted at the Michael E. DeBakey Veterans Affairs Medical Center and its satellite clinics and was approved by the local institutional review board. We used a mixed methods approach analogous to root cause analysis [20] to uncover potential workflow or technical reasons for lack of clinician response to positive FOBT results. We conducted eleven semi-structured interviews with key informants from the laboratory, primary care, and information technology sections to gather details related to FOBT alert generation, transmission, and receipt. Concurrently, we obtained quantitative data to track the alert receipt and follow-up actions by providers.

Clinicians in the VA health care system receive notifications of high-priority information such as abnormal test results in a "View Alert" window of the electronic health record. To understand the technical issues surrounding electronic communication, we analyzed and mapped the associated system-level processes involved. We discovered that the FOBT alert communication system is driven by an underlying component of the electronic health record that continually monitors test order and result entry. Alerts are automatically generated and recipients selected based on a set of predefined rules and parameters. For instance, entry of a test result such as positive FOBT (which was pre-determined to be a high-priority test result) will generate an automated notification to one or more clinicians. The proper recipients for this notification are chosen based on the setting of certain system parameters. After delivery to recipients, alerts stay active in the clinician's inbox up to two weeks, or until acknowledged.

Using the alert tracking system of the electronic health record, we identified all positive FOBT alerts transmitted daily during our study period. Approximately three weeks after alert generation, a trained physician reviewed the electronic health record for evidence of timely FOBT follow-up using a standardized data collection form that had been pilot tested in previous work [19]. Any documented response to the FOBT, such as colonoscopy referral, patient notification, or mention of exclusion criteria for colonoscopy, was considered timely follow-up. If no follow-up action was documented, an additional investigator confirmed the findings and called the ordering clinician (usually the primary care practitioner-PCP). If the clinician gave convincing information to support any undocumented actions, we considered this response as evidence of timely follow-up as well. We also recorded clinicians' comments and actions.

Following a trail of positive FOBTs that were found to have lack of timely follow-up, we used purposeful sampling and snowball techniques to identify our study subjects [21]. We initially purposefully sampled three PCPs whose FOBT results were found on chart review to have not received follow-up. Information from these PCPs led to further interviews with 1 additional provider (a subspecialist) and representatives that were involved with FOBT performance (laboratory personnel) and FOBT reporting (laboratory and Information Technology personnel). Additionally, 3 institutional representatives from leadership and administration that oversee workflow related to FOBT results were also interviewed.

**Figure 1:**
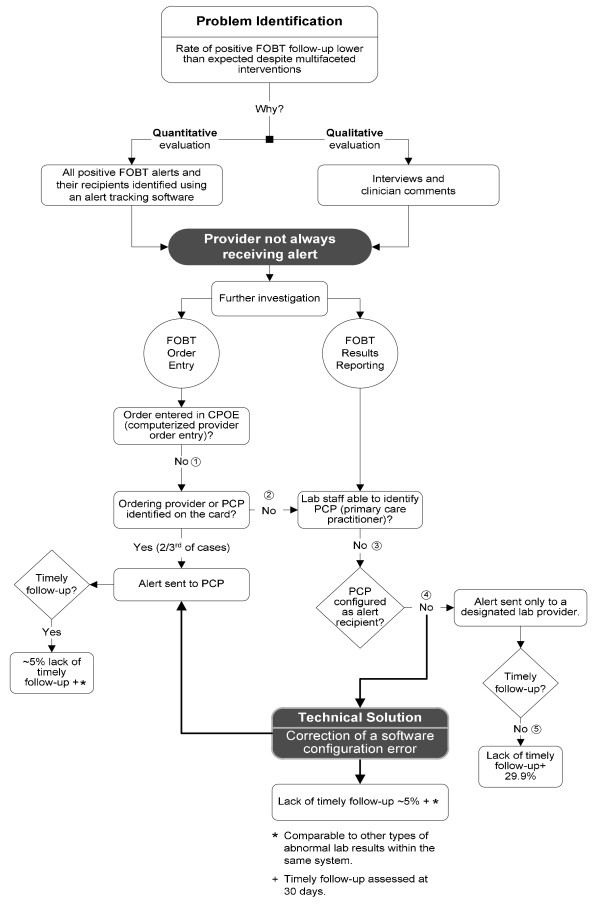
**Workflow and technical analysis to investigate root cause of high rates of non- response to positive FOBT (fecal occult blood test)**. Contributing steps 1-5 identified.

We gathered data from interviews, clinicians' comments and FOBT tracking to uncover reasons for lack of timely follow-up (Figure 1). Using themes generated from this data, we found that five steps contributed to the problem, one of which was a software configuration error in the alert communication system. The latter step was the most significant one in the final common pathway and most amenable to a systems based intervention to improve communication and follow-up of positive FOBTs. To assess effect of the intervention we implemented, we compared rates of follow-up of positive FOBTs pre- and post-intervention using a Z test of two proportions.

## Results

### Problem Identification

Data from PCP interviews and reported comments suggested that PCPs were not receiving positive FOBT alerts consistently, leading us to further investigate the processes associated with FOBT alert generation. Workflow analysis revealed that a large number of patients who are given FOBT cards never return them to the lab for processing, and therefore an order for the test (through a computerized order-entry system) is only placed upon receipt of the card by the lab. However, in the absence of a provider-generated computerized order, the ordering provider is not easily identifiable unless written on the card. Because lab technicians use a different order-entry system, it is difficult for them to identify the ordering provider (and hence the primary recipient of the alert).

Further analysis of alert generation revealed that, regardless of an identifiable ordering provider in the system, the alert management software is designed to communicate all high priority alerts to the PCP as long as a primary alert recipient is identified. We discovered that in positive FOBTs where the ordering provider was not identified, a laboratory staff member served as the designated "ordering" provider, i.e. the primary recipient for the alert. This workaround (nonstandard procedures typically used because of deficiencies in system or workflow design) [22] was intended to enable the completion of the order and subsequent transmission of any alert generated to the patient's PCP; a fail-safe or safety-net mechanism designed to prevent loss of FOBT follow-up.

However, additional technical analysis of the alert tracking data revealed that in all cases where the designated lab provider was alerted as the "ordering" provider, there was no concomitant alert transmission to the PCP. Thus, only the lab provider was receiving the positive FOBT results and had no knowledge of this technical problem. We categorized such alerts as designated lab provider alerts and found that lack of timely follow-up was much more prevalent in this subgroup of alerts (29.9% vs. 4.5% in non-designated lab provider alerts).

### Intervention

We surmised that a lack of PCP awareness (in over a third of cases with positive FOBTs) contributed substantially to the prevalence of FOBT results with no documented follow-up, and that a software configuration error was the root of the problem. Once the electronic health record determines the need to generate an alert, proper recipients are selected based on their relationship to the patient (i.e., ordering provider, PCP, etc.). We found an improper configuration of the parameter that defines these default recipients, such that the PCP was not selected as a recipient for designated lab provider alerts (i.e. when PCPs were not listed as ordering providers). However, we could not determine when and how this error occurred in the system. Nevertheless, we posited that a problem-specific fix of this incorrect software configuration would reduce the risk of loss of follow-up for these alerts. The solution to this problem, an addition of a code to link patients to their PCP for tests ordered by others, was implemented on November 28, 2008 (date of intervention).

### Evaluation

**Figure 2:**
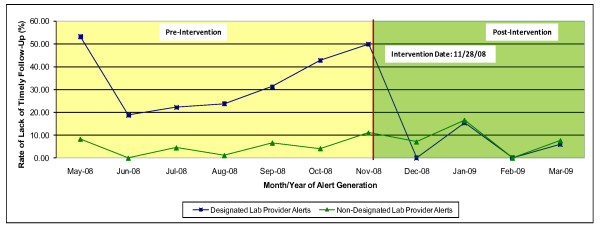
**Follow Up of Positive Fecal Occult Blood Tests Pre- and Post-Intervention to Correct Software Configuration Error**.

We reviewed 360 alerts (117 designated lab provider alerts) pre-intervention and 130 alerts (55 designated lab provider alerts) post-intervention. Figure 2 shows the monthly prevalence of designated lab provider and non- designated lab provider alerts without timely follow-up pre- and post-intervention. Pre-intervention, lack of timely follow-up was observed for 29.9% of the designated lab provider alerts and 4.5% of non- designated lab provider alerts group. However, in the time period following the intervention, the percentage of designated lab provider alerts without timely follow-up decreased to 5.4% (p < 0.01) and was not statistically significantly different from that of non- designated lab provider alerts (6.6%; p = 0.9). This rate decrease occurred immediately following the intervention and remained stable (i.e. lower than pre-intervention levels) in the subsequent four months (Figure 2). Post-intervention tracking data confirmed that alerts assigned to the designated lab provider were now also being transmitted to the patient's PCP.

## Discussion

We investigated reasons why follow-up actions on a large proportion of positive FOBT results that needed a diagnostic colonoscopy were not documented by clinicians despite the use of a system to electronically communicate positive results. In addition to order-entry workarounds in the electronic health record, we discovered that the communication system intended to alert PCPs of positive FOBT results was not configured correctly, leading to certain situations in which PCPs never received the test result. Upon correction of the software configuration error, the percentage of positive FOBT results lacking follow-up were dramatically reduced. Although the rate did not drop to zero, it was comparable to the rate of lack of timely follow-up we found for other types of non-life threatening, high-priority lab notifications in the same system [23]. Our findings suggest that communication of cancer-related test results in the electronic health record must be monitored to avoid compromising the promise of cancer screening programs.

Of the over 800 patients each year who have positive FOBTs at our institution, about 10-15% of them are eventually diagnosed with some form of colon disease (including cancer). None of the patients in our study had any delay in cancer diagnosis or related harm. Although it is possible that follow-up may have occurred beyond our 30 day "timely response" window had we not intervened, previous work suggests that many of these findings would ultimately never be followed-up [10,11,19]. Thus, our seemingly small intervention could potentially have a large impact on decreasing time to referral for colonoscopy, thereby reducing the risk of a missed or delayed diagnosis of colorectal cancer, a common reason for ambulatory malpractice claims [24-26]. Previous literature has highlighted the need for system-based interventions to improve follow-up of positive cancer screens and our study is one of few that contributes to this body of knowledge [6].

Our findings also highlight how electronic health record use can have dramatic effects on follow-up care of patients. Electronic health records have potential to address the fragmented and discontinuous care that usually characterizes care in the outpatient setting. Critical information flow between different practitioners, settings and systems of care is essential to high quality care. Through good decision support systems, transmission of information to the right provider at the right time is within the reach of integrated electronic health records. However, as we find, electronic health record use must take into account the effect electronic communication will have on workflow and vice versa. Not doing this correctly would lead to circumstances that reduce the situational awareness of providers and perhaps other unintended adverse effects.

A limitation of our study was a lack of comparable data from other VA or non-VA facilities. However, our work illustrates how electronic test result communication systems are susceptible to errors that may limit their intended outcomes. Furthermore, it should be noted that other VA investigators [10,11] have demonstrated high rates of lack of positive FOBT follow-up, so it is possible that this problem exists at other VA sites. We are currently investigating whether this problem exists in other VA facilities or if this was an isolated event. Additionally, in this study we did not address many other systems issues that should be considered to address follow-up of abnormal test results in addition to provider, technology and work-flow. In our work, we are now using a socio-technical model that accounts for many other systems issues beyond the responsible provider, including the role of organizations and policies and procedures to address monitoring of abnormal test results [27]. For instance, an institutional policy that all FOBTs are ordered through computerized order entry would be another intervention to address this area. In our future work, we will propose multifaceted solutions to address the many complex issues related to abnormal test result follow-up.

Although electronic health records likely offer many benefits over paper-based systems for improving communication of abnormal cancer screening results [17], our findings highlight the need to account for inherent complexities of clinical practice. This complexity may introduce circumstances requiring special attention to EHR workflow to prevent loss of follow-up of important clinical information. In our setting, several workarounds of the FOBT ordering and reporting process resulted in disruption of the normal electronic health record workflow, creating a reliance on a secondary PCP notification system, which was not functioning as intended. The challenge of recognizing these complexities and their effects underscores the need for continuous monitoring of key electronic health record features that may impact safety. The work described here was a direct result of quality assurance work that is highly regarded in the VA health care system. Other institutions could use our methods to track the effectiveness of electronic communication. However, quality monitoring procedures such as used by the VA to ensure system safety must also be used to identify red flags that would lead to similar future investigations. Without the safeguards used by the VA, the problems related to test result communication may go undetected.

Health care systems should aim to achieve a high reliability for tracking delivery of abnormal cancer screening results. An example viewed as an ideal model for tracking systems is that of FedEx, which is considered to have 99.6% tracking reliability for its packages [28]. To achieve such high tracking reliability would not only require implementation of comprehensive technology-based systems for communication, but also formal policies and procedures regarding their use [28]. The test result communication system evaluated in our study addressed several criteria [28] for effective critical results reporting systems, such as computerized tracking and back-up procedures. However, to achieve tracking comparable to other industries, cancer screening programs should continuously monitor and oversee the timely delivery of positive cancer screening results to the right clinicians. For example, we recommend that cancer screening programs using electronic health record systems should develop and monitor multiple metrics of performance of automated communication processes. Failure to implement such monitoring systems could lead to sub-optimal screening success, which may otherwise be difficult if not impossible to trace.

## Conclusion

In conclusion, we believe that electronic health records are beneficial in communicating abnormal cancer screening results to clinicians and will improve their follow-up care; however, we cannot assume that electronic communication is always working exactly as expected especially when workarounds are used. To achieve the most benefits of cancer screening programs, robust monitoring systems are necessary in electronic health record systems to ensure that abnormal cancer screening results are being delivered to the correct providers in a timely manner.

## Competing interests

The authors declare that they have no competing interests.

## Authors' contributions

HS conceived of the study, participated in its design and coordination, and drafted the manuscript. LW participated in the statistical analysis and qualitative data collection. LP participated in drafting and editing the manuscript as well as the design of the study. MS was involved in the qualitative data collection and analysis. BR was involved in qualitative data collection with Information Technology personnel and drafting the results of the manuscript. DE participated in the coordination of the study as well as qualitative data collection. DS participated in data analysis and provided edits to the final manuscript. All authors read and approved the final manuscript.

## Authors' information

The study was supported by an NIH K23 career development award (K23CA125585) to Dr. Singh, the VA National Center of Patient Safety, and in part by the Houston VA HSR&D Center of Excellence (HFP90-020). These sources had no role in the design and conduct of the study; collection, management, analysis, and interpretation of the data; and the preparation, review, or approval of the manuscript. The views expressed in this article are those of the authors and do not necessarily represent the views of the Department of Veterans Affairs.

## Pre-publication history

The pre-publication history for this paper can be accessed here:

http://www.biomedcentral.com/1472-6947/9/49/prepub
